# Einsatz von Virtual Reality in der HNO-Lehre: eine Alternative zum konventionellen Anatomiemodell

**DOI:** 10.1007/s00106-022-01252-z

**Published:** 2022-12-07

**Authors:** P. von Schnakenburg, S. Heermann, J. Kromeier, C. Offergeld

**Affiliations:** 1 Klinik für Hals‑, Nasen- und Ohrenheilkunde, Medizinische Fakultät, Universitätsklinikum Freiburg, Albert-Ludwigs-Universität Freiburg, 79085 Freiburg, Deutschland; 2 Institut für Anatomie und Zellbiologie, Abteilung Molekulare Embryologie, Albert-Ludwigs-Universität Freiburg, Freiburg, Deutschland; 3 Klinik für Diagnostische Radiologie, Kinderradiologie, Neuroradiologie und Interventionelle Radiologie, St. Josefskrankenhaus, Artemed Kliniken Freiburg GmbH, Freiburg, Deutschland

**Keywords:** Digitalisierte Lehre, Medizinstudenten, Akzeptanz, Mittelohranatomie, Digitale Transformation, Digitalised teaching, Medical students, Acceptance, Middle ear anatomy, Digital transformation

## Abstract

**Hintergrund:**

Die Beurteilung des Mittelohrs erfordert ein komplexes dreidimensionales Verständnis, dessen Vermittlung für die curriculare Lehre ebenso wichtig ist wie für die ärztliche Weiterbildung.

**Zielsetzung:**

Überprüft wurde, inwieweit Virtual Reality (VR) als Alternative zu konventionellen Lehrmethoden in der Vermittlung von Inhalten der Anatomie, Physiologie und Pathologie zum Einsatz kommen kann. Zielsetzung ist die Evaluation einer VR-gestützten Lehrmethode im Vergleich zur konventionellen Lehre am anatomischen Modell.

**Methodik:**

Die Studie wurde als zweiarmige prospektive Single-Center-Studie im Sommersemester 2021 an der Universitätsklinik Freiburg durchgeführt. Für ein Modul zum Thema Mittelohr wurden 177 Studierende randomisiert in eine Kontroll- und Studiengruppe eingeteilt. Vorab wurden demografische Daten abgefragt sowie eine quantitative Evaluation hinsichtlich Kompetenz und persönlicher Haltung erhoben. Nach Bearbeitung der Modelle wurden die Gruppen formativ geprüft und die Ergebnisse vergleichend untersucht. Abschließend wurde durch ein Crossover der Modelle eine qualitative Evaluation der Modelle im Vergleich ermöglicht und eine erneute quantitative Evaluation durchgeführt.

**Ergebnisse:**

In der formativen Prüfung konnte kein signifikanter Unterschied zwischen den Gruppen nachgewiesen werden. Die Evaluationen konnten eine gesteigerte Selbsteinschätzung der Wissenskompetenz, eine tendenziell ausgesprochen positive Haltung zur VR-Methode nach Kursabschluss sowie generell vorteilhafte subjektive Aspekte des VR-Modells aufweisen. Zudem zeigte sich ein positiver Effekt und ein positives Meinungsbild für die Vermittlung anatomischer Inhalte.

**Schlussfolgerung:**

Der Einsatz von VR eignet sich als Alternative zu konventionellen Lehrmethoden in der curricularen HNO-Lehre. Die Ergebnisse zeigen bereits aktuell eine Gleichwertigkeit der VR und lassen ein großes Potenzial dieser Methode für zukünftige Lehraufgaben erwarten.

**Zusatzmaterial online:**

Die Online-Version des Beitrages 10.1007/s00106-022-01252-z enthält eine weitere Abbildung: Fragebogen – Q2.

## Abstract (en)

**Background:**

Assessment of the middle ear requires a complex three-dimensional understanding, the teaching of which is just as important for curricular teaching as for further medical training.

**Objective:**

The extent to which virtual reality (VR) can be used as an alternative to conventional educational methods for teaching anatomy, physiology, and pathology was examined. The objective was to evaluate a VR-supported teaching method in comparison to conventional learning on an anatomic model.

**Methods:**

The study was conducted as a prospective two-arm single-center trial in the summer semester of 2021 at the University Hospital of Freiburg. During a seminar on the topic of the middle ear, 177 students were randomly assigned to either a control or a study group. Demographic data were collected in advance and a quantitative evaluation regarding competence and personal attitude was performed. After processing the models, the groups were formatively tested and the results were analyzed comparatively. Ultimately, crossover of the models enabled a qualitative comparative evaluation of the models and a renewed quantitative evaluation was carried out.

**Results:**

No significant differences between the groups were identified in the formative testing. The evaluations demonstrated increased self-assessment of knowledge competence, a tendency towards a highly positive attitude towards the VR method after completion of the course, and generally beneficial subjective aspects of the VR model. In addition, there was a positive effect and a positive perception of the delivery of anatomic content.

**Conclusion:**

Use of VR is suitable as an alternative to conventional teaching methods in curricular ENT teaching. The current evidence demonstrates the equality of VR and indicates its great potential for future educational tasks.

## Hintergrund

Die SARS-CoV-2-Pandemie prägt seit März 2020 das tägliche Leben an den Universitäten [21]. Die Lehrstrukturen mussten unter diesen Bedingungen angepasst werden, sodass nach anfänglich nicht unerheblichen Problemen die digitale Transformation große Fortschritte vorweisen kann [17]. Zahlreiche neue Lern- und Lehrmethoden sowie neue didaktische Ansätze fanden Einzug in den curricularen Alltag, so auch in der HNO-Heilkunde [23]. Studien zeigen, dass die Vermittlung digitaler Kompetenzen in der medizinischen Ausbildung nach vorübergehender Vernachlässigung mittlerweile deutlich intensiver in Lehrkonzepte von Fachdisziplinen integriert wird [5, 18, 19]. In der Freiburger HNO-Heilkunde wurde schon präpandemisch im Rahmen der „digitalen Transformation“ an der Implementierung digitaler Strukturen gearbeitet, wie ein als eigenständige Webpage angebotenes HNO-Lernprogramm belegt [6]. Diese neue Lehrplattform zeigte eine hohe Akzeptanz bei den Studierenden sowie vielversprechende Verbesserungen in der Wissensvermittlung [12]. Der Themenkomplex des Mittelohrs wird beispielsweise mit primärem Fokus auf einem anatomischen Verständnis und HNO-spezifischen Aspekten mittels dreidimensionalen Darstellungen eindrücklich behandelt. Im Gegensatz dazu können bislang praktizierte Lehrangebote (z. B. Atlanten) die anatomische Dreidimensionalität von mikroskopischen Strukturen nur sehr eingeschränkt vermitteln. Die fortschreitende Digitalisierung der Lehre bietet hierbei nun neue Technologien und Möglichkeiten (z. B. 3‑D-Modelle) [15]. Augmented Reality (AR) und VR erschaffen eine alternative, digitalisierte Realität und ermöglichen es, virtuelle Strukturen im Raum zu betrachten [29], was insbesondere für das Verständnis von Verhältnissen dreidimensionaler Strukturen vorteilhaft sein kann [14]. Da die neuartige Technik außerdem einen positiven Effekt auf Lernmotivation und -erfahrung zu haben scheint, soll die Frage geklärt werden, ob sich mittels VR Vorteile beim Erlernen komplexer anatomischer Strukturen in der HNO-Lehre ergeben [26].

## Zielsetzung

Ziel der Studie war die Quantifizierung von Effizienz und Akzeptanz eines VR-Modells des Mittelohrs zur Wissensvermittlung komplexer dreidimensionaler Strukturen und ihrer Topografie. Es wurde erwartet, dass unter kontrollierten und randomisierten Konditionen der Einsatz des VR-Modells zu Vermittlung der Mittelohr-Anatomie den konventionellen Plastikmodellen hinsichtlich der objektiv überprüfbaren Wissensvermittlung überlegen ist. Zudem sollte das subjektive Lernempfinden der Studierenden analysiert werden.

## Methodik

### Stichprobe und Untersuchungsablauf

Die Studie wurde im zweiarmigen prospektiven Design im Sommersemester 2021 mit 177 Studierenden an der HNO-Klinik des Uniklinikum Freiburg als Single-Center-Studie durchgeführt. Nach regulärem Semesterplan belegen die Studierenden den Kurs des Fachbereich HNO im 7. oder 8. Fachsemester. Den je zweiwöchigen Blockkurs durchliefen die Studierenden über das Semester verteilt nacheinander in sieben Gruppen mit jeweils 22–30 Personen. Aufgrund der pandemiebedingten Hygienevorschriften für Präsenzveranstaltungen wurden die Studierenden zur Kontaktminimierung in Zweiergruppen eingeteilt.

**Figure 1:**
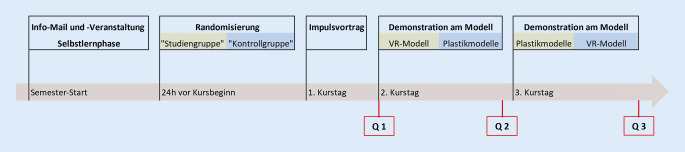

Die Abb. 1 gibt eine Übersicht des Studienablaufs. Zu Beginn des Semesters wurde eine Informationsveranstaltung über den Ablauf der Studie angeboten. In der zweiten Woche der jeweiligen Blockkurse sollte ein an drei aufeinanderfolgenden Kurstagen stattfindendes Seminar zum Thema Mittelohr absolviert werden. Die Studierenden konnten sich hierfür im Vorfeld mithilfe eines E‑Learning-Moduls auf der klinikeigenen Online-Plattform vorbereiten [6, 12]. Die Anwendung einer ersten Selbstlernphase zur Vermittlung von Faktenwissen als Grundlage vor der eigentlichen Präsenzphase folgt dem Konzept des „flipped classroom“ [4, 27].

**Figure 2:**
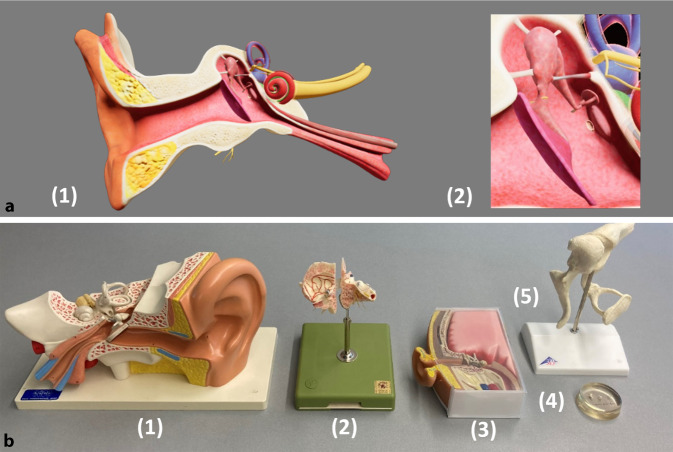

Am ersten der drei Tage gab ein Online-Impulsvortrag allen Studierenden einen tiefergehenden Einblick in den Themenkomplex „Mittelohr“. An Tag zwei und drei folgten Präsenzkurse bestehend aus jeweils drei Stationen mit praktischen Übungen oder der inhaltlichen Auseinandersetzung mit der Thematik. Eine dieser Stationen diente an beiden Tagen der Vertiefung der komplexen Topografie und Anatomie des Mittelohrs durch ein Studium an Modellen. An dieser Station wurde die Studie zum VR-Modell durchgeführt. Hierfür wurden die Studierenden in ihrer Zweiergruppe EDV-basiert randomisiert, in zwei Gruppen eingeteilt und nach der Ausrichtung für den primären Endpunkt benannt. Eine Gruppe wurde als Studiengruppe (SG) bestimmt, die das VR-Modell bearbeiten durfte (*n* = 88). Die Studierenden der anderen Gruppe dienten als Kontrollgruppe (KG) und sollten ihr Wissen an den konventionellen Plastikmodellen vertiefen (*n* = 89). Die Abb. 2 zeigt eine Ansicht der jeweiligen Modelle. Die Betreuung der Studierenden erfolgte durch didaktisch und fachlich geschulte studentische Tutoren (sog. Peers) sowie durch eine ärztliche Aufsicht.

Vor Beginn des ersten Präsenzseminars wurde der erste Fragebogen (Q 1) bearbeitet. Im Anschluss erfolgte die Zuweisung zu den genannten Gruppen. Die entsprechenden Modelle wurden den Zweiergruppen für 15 min zur Verfügung gestellt. Beim VR-Einsatz kommt ein bereits publizierter standardisierter Durchlauf durch das Modell zum Einsatz [20], der es den Studierenden ermöglicht, einen umfassenden und orientierenden Einblick in das Modell zu bekommen. Es wurden zwei VR-Geräte (OculusRift S, Fa. Oculus VR, Menlo Park, CA, USA) zur Verfügung gestellt, sodass den Zweiergruppen eine parallele Bearbeitung ermöglicht werden konnte. Es galt, sich die komplexe Anatomie des Mittelohrs anhand eines strukturierten Durchlaufens des Modells zu erschließen. Dabei sollte der Weg des Schalls wortwörtlich „verfolgt“ werden. Ein Eintauchen in die Strukturen macht es dabei möglich, diese auszublenden. Text, Ton oder Animationen sind in dem Modell nicht implementiert. Die Betreuer der Station standen für Fragen zu Verfügung und unterstützten bei der Orientierung im Modell. Nach der 15-minütigen Bearbeitung der jeweiligen Lehrmethode folgte ein zweiter Fragebogen (Q 2) zum Abschluss des primären Endpunkts. Der dritte Tag erfolgte erneut in Präsenz, wobei die Gruppen nun getauscht wurden, sodass auch die KG Erfahrungen mit dem VR-Modell sammeln konnte, während diesmal die SG die Plastikmodelle präsentiert bekam. Beide Gruppen hatten nun die unterschiedlichen Lehrmethoden bearbeitet und konnten in einem abschließenden dritten Fragebogen (Q 3) evaluierende und vergleichende Aussagen treffen. Alle Fragebogen wurden pseudonymisiert, um eine Verknüpfung der drei Fragebögen möglich zu machen. Nach der Zusammenführung der Daten wurden diese anonymisiert weiterverwendet.

Die Tab. 1 gibt eine Übersicht über die Inhalte der Fragebögen Q 1–3. Der primäre Endpunkt wurde durch den Fragebogen Q 2 erfasst, in dem 10 Multiple-Choice-Fragen das Wissen inhaltlich überprüften (s. Supplementary Information). Zur Evaluation des Kurses und der jeweiligen Modelle kam eine quantitative und eine qualitative Evaluation zum Einsatz. Die quantitative Evaluation zielte auf eine Quantifizierung des subjektiven Lernfortschritts über das Seminar sowie der Veränderung der generellen Haltung zum Einsatz der neuen Technik ab. Hierfür wurde in allen drei Fragebögen (Q1–3) die Selbsteinschätzung der Kompetenz zum Themenkomplex (1 = sehr gering, 3 = neutral, 5 = sehr hoch) sowie in den Fragebögen Q 1 und Q 3 die persönliche Haltung zum Einsatz der VR-Methodik (1 = sehr negativ, 3 = neutral, 5 = sehr positiv) in Form von 5‑stufigen Likert-Skalen erfragt. Die qualitative Evaluation diente dem subjektiven Vergleich der beiden Methoden (Plastikmodelle und VR-Modell) für unterschiedliche Qualitäten und enthielt zehn zu bewertende Aussagen ebenfalls mit 5‑stufigen Likert-Skalen (1 = stimme vollständig zu, 3 = lehne weder ab, noch stimme zu, 5 = lehne vollständig ab).

**Table 1:** 

Q 1	Q 2	Q 3
*Demografische Daten*	*Kompetenzabfrage*	*Qualitative Evaluation*
Altersgruppe Geschlecht Nutzung der Lehrangebote (Vortrag und E‑Learning) Erfahrung mit Virtual Reality (VR)	10 Inhaltliche Multiple-Choice-Fragen 10 zu erreichende Punkte	Subjektives Lernerlebnis im rückblickenden Vergleich der Verfahren 10 zu bewertende Aussagen
*Quantitative Evaluation*	*Quantitative Evaluation*	*Quantitative Evaluation*
Selbsteinschätzung der Kompetenz zum Themenkomplex, persönliche Haltung zum Einsatz von VR	Selbsteinschätzung der Kompetenz zum Themenkomplex	Selbsteinschätzung der Kompetenz zum Themenkomplex, persönliche Haltung zum Einsatz von VR

### Statistische Auswertung

Als Primärauswertung der Kompetenzabfrage wurde der Mann-Whitney-U-Test durchgeführt. Deskriptiv wurde darüber hinaus die Mittelwertdifferenz zwischen beiden Gruppen mit zweiseitigem 95%-Konfidenzintervall (95%-KI) geschätzt – eine Mittelwertdifferenz von mindestens zwei Punkten wurde als relevant angesehen. Sekundäre Endpunkte wurden deskriptiv ausgewertet und in explorativen Regressionsmodellen sowie mittels gepaarten und ungepaarten T‑Tests untersucht. *p*-Werte < 0,05 (5 %) wurden dabei als signifikant betrachtet. Demografische und andere Ausgangsdaten wurden deskriptiv aufgefasst.

Die Datenanalyse erfolgte mittels des Statistikprogramm GraphPad Prism (Version 9.3.0 für Windows, Fa. GraphPad Software, San Diego/CA, USA). Die Erstellung der Tabellen erfolgte mit Excel bzw. Word (Fa. Microsoft 2010, Redmond/WA, USA).

## Ergebnisse

Von den 177 Teilnehmern haben alle den Fragebogen Q 2 zum primären Endpunkt ausgefüllt. Allerdings fehlten bei sieben Personen Teile oder der komplette Q 1 und bei vier Personen der Q 3, sodass sie für die sekundären Endpunkte ausgeschlossen wurden. In die quantitative Evaluation konnten daher 169 Teilnehmer einbezogen werden und 173 in die qualitative Evaluation.

### Formative Prüfung (primärer Endpunkt)

**Figure 3:**
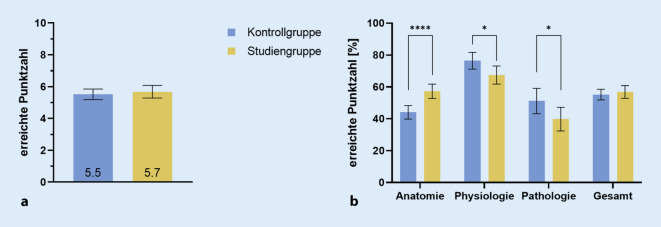

In der Kompetenzabfrage erreichte die KG bei den 10 zu beantwortenden Fragen im Mittel 55,2 % (95%-KI: 51,8–58,5 %), die SG hingegen erreichte 56,8 % (95%-KI: 52,9–60,8 %) der Gesamtpunktzahl. Es zeigt sich eine Mittelwertdifferenz von ∆ 1,6 % (95%-KI: −3,5 bis 6,8 %) beziehungsweise von ∆ 0,2 bei 10 erreichbaren Punkten (95%-KI: −0,4 bis 0,7) zugunsten der VR-Gruppe (Abb. 3b). Statistische Analysen zum Vergleich der erbrachten Leistung konnten keinen Unterschied zwischen den Gruppen nachweisen (*p* = 0,49).

Die Fragen lassen sich inhaltlich in die drei Themenbereiche Anatomie, Physiologie und Pathologie unterteilen (Abb. 3c). Der Bereich der Anatomie wurde durch die ersten fünf Fragen abgedeckt. Die KG erreichte hier ein Mittel von 2,2 (95%-KI: 2,0–2,4) von 5 Punkten, die SG hingegen eine mittlere Punktzahl von 2,9 (95%-KI: 2,6–3,1). Von den drei Fragen zu Physiologie wurden in der KG im Mittel 2,3 (95%-KI: 2,1–2,4) und in der SG 2,0 (95%-KI: 1,9–2,2) von 3 Punkten erreicht. Die Pathologie wurde mit zwei Fragen behandelt, wobei die mittlere erreichte Punktzahl in der KG bei 1,0 (95%-KI: 0,9–1,2) und in der SG bei 0,8 (95%-KI: 0,6–0,9) von 2 Punkten lag. Analysen zum Vergleich der von SG und KG erbrachten Punkte zeigten in allen drei Bereichen signifikante Unterschiede: in der Anatomie ∆ 0,7 Punkte (95%-KI: 0,4–1,0; *p* < 0,0001) zugunsten der SG; in der Physiologie ∆ 0,3 Punkte (95%-KI: 0,0–0,4; *p* = 0,0215) und in der Pathologie ∆ 0,2 Punkte (95%-KI: 0,0–0,4; *p* = 0,0390) zugunsten der KG.

### Kompetenzzuwachs (quantitative Evaluation)

**Figure 4:**
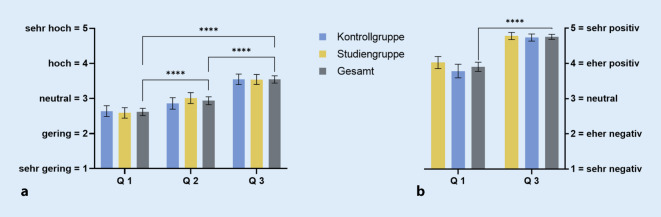

Zu Beginn gaben die Teilnehmer eine mittlere Selbsteinschätzung von 2,6 (± 0,7) von 5 Punkten zum eigenen Wissen ab (Abb. 4a). Im Verlauf des Kurses steigerte sich die mittlere Angabe in der KG zunächst auf 2,9 Punkte (95%-KI: 2,7–3,0) und in der SG auf 3,0 Punkte (95%-KI: 2,9–3,2). Zwischen den Gruppen zeigt sich hier kein statistisch relevanter Unterschied (∆ 0,15; −0,073 bis 0,38; *p* = 0,1846). Am Ende des Kurses wurde ein gemitteltes Wissen von 3,5 Punkten (95%-KI: 3,4–3,6) angeben.

Bei statistischer Analyse zeigen sich die Anstiege von Q 1 auf Q 2 mit ∆ 0,3 Punkten (95%-KI: 0,22–0,42; *p* < 0,0001), von Q 2 auf Q 3 mit ∆ 0,6 Punkten (95%-KI: 0,5–0,7; *p* < 0,0001) sowie der Gesamtanstieg von Q 1 auf Q 3 mit ∆ 0,9 Punkten (95%-KI: 0,81–1,1; *p* < 0,0001) stark signifikant.

### Persönliche Haltung (quantitative Evaluation)

Zur Persönlichen Haltung zum Einsatz der VR-Methode in der Lehre gaben die Studierenden vor dem Seminar im Mittel 3,9 (±0,8) von 5 Punkten an (Abb. 4b). Nach Abschluss des dritten Kurstages ergab sich ein Mittel von 4,8 Punkten (95%-KI: 4,7–4,8). Dieser Anstieg um ∆ 0,9 Punkte erwies sich als statistisch signifikant (95%-KI: 0,7–1,0; *p* < 0,0001).

### Subjektive Einordnung (qualitative Evaluation)

Für diese Diskussion werden hier nur 8 der 10 zu bewertenden Aussagen aufgeführt. Die beiden Aussagen „Die VR stellt vor allem ein ‚Gadget‘ dar: Nice to have, aber für den Lernprozess verzichtbar“ und „Ich kann mir NICHT vorstellen, dass VR für die Vermittlung von praktischen Kompetenzen gegenüber den Plastikmodellen einen Mehrwert hat“ wurden als Teil eines vorab entworfenen, umfassenden Fragenportfolios mit abgefragt, zeigten aber im Verlauf für die Gesamtkonstellation wenig Relevanz.

In einer an *Aussage 7* angeknüpften Freitext-Frage zu etwaigen körperlichen Beeinträchtigungen oder technischen Überforderungen gaben einige Studierende leichte Kopfschmerzen (x = 2) sowie Übelkeit oder Schwindel (x = 10; „motion sickness“) an.

## Diskussion

Der Nationale Kompetenzbasierte Lernzielkatalog Medizin (NKLM 2.0) beschreibt die Lernziele und das Kerncurriculum des neu ausgerichteten humanmedizinischen Studiums [15]. Zu dem Themenkomplex „Mittelohr“ setzt der NKLM 2.0 ein Handlungs- und Begründungswissen entsprechend der Kompetenzstufe 2 voraus (vgl. VII. 1a-18.4.1). Die Studierenden sollen demnach in der Lage sein, wesentliche Strukturen und Prozesse zu erklären und dabei u. a. den Aufbau und die Funktion des Mittelohrs sowie den klinisch-wissenschaftlichen Kontext zu beschreiben. Dies setzt eine eigenverantwortliche thematische Vorbereitung der Studierenden voraus mit der hieraus resultierenden Notwendigkeit, die Wissensvermittlung so effektiv wie möglich zu gestalten. Effektives Lernen definiert sich in erheblichem Maße durch praktische Prozeduren, deren Wiederholungen und einem Verständnis für die jeweilige Thematik [9, 22]. Dies bedingt aber auch, dass sowohl Effektivität als auch Qualität der angewandten Lehrmethoden diesem erhöhten Anspruch gerecht werden sollten.

Hierfür bringt die „digitale Transformation“, die auch die medizinische Hochschullehre inkludiert, viel Potenzial mit sich. Die Entwicklung virtueller Realitäten erlauben so neue Möglichkeiten der Wissensvermittlung. Es handelt sich hierbei um Präsentationsformen, die dazu geeignet sind, abstrakte räumliche Informationen, die den Augen des Betrachters unter normalen Umständen nicht unmittelbar zugänglich sind, erfahrbar zu machen [24]. Es können aber auch weniger spektakuläre, klinische Routineuntersuchungen mithilfe der VR patientenunabhängig zu Ausbildungszwecken eingesetzt werden [1], was in Zeiten der Corona-Pandemie eine enorme Hilfe bei der Aufrechterhaltung von Präsenzunterricht war und ist [17]. Die Maximalvariante einer solchen realitätsnahen Simulation wird als „immersive patient simulator“ (IPS) bezeichnet [8].

**Figure 5:**
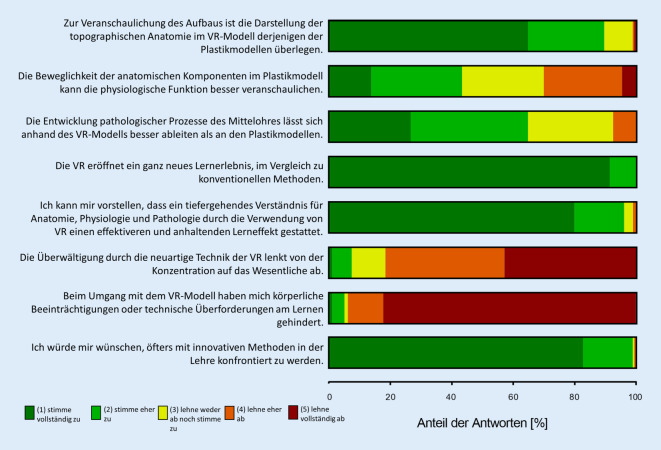

Unsere Ergebnisse einer vergleichenden formativen Prüfung ließen keinen signifikanten Unterschied in der Kompetenzvermittlung zwischen dem konventionellen und dem virtuellen Modell feststellen (Abb. 3a). Die Hypothese einer Überlegenheit des VR-Modells konnte somit aktuell nicht belegt werden. Gleichwohl steht zu bedenken, dass das VR-Modell sich auf Anhieb als gleichwertige und valide Alternative zur bislang führenden Lehrmethode anhand der Ergebnisse präsentierte. Da im vorliegenden VR-Modell noch längst nicht alle Kapazitäten ausgeschöpft sind, darf spekuliert werden, wie ausgeprägt eine weitere Steigerung der subjektiven Zustimmung bzw. des Lernzuwachses der Benutzer bei einer verbesserten Variante aussehen könnte. So sollen zukünftig Beschriftungen der einzelnen möglicherweise hervorgehobenen Elemente implementiert werden und auch einzelne Komponenten ausgewählt und individuell im Raum betrachtet werden können. Unsere Ergebnisse zeigen bereits jetzt, insbesondere in der Vermittlung anatomischer Inhalte, Hinweise auf einen positiven Effekt bei der Verwendung des VR-Modells (Abb. 3b). Diese Tendenz wird eindrucksvoll in der vergleichenden qualitativen Evaluation der Studierenden (Abb. 5) untermauert. Dort wird der Vorteil des VR-Modells gegenüber den konventionellen Modellen ebenfalls besonders in der Vermittlung der anatomischen Komponenten angegeben (*Aussage 1*). Auch die Entwicklung pathologischer Prozesse sei eher auf der Seite des VR-Modells einfacher nachzuvollziehen (*Aussage 3*). Alleinig bei der Bewertung der Veranschaulichung physiologischer Aspekte zeigt sich ein eher neutrales Bild (*Aussage 2*). Dies steht im Einklang mit dem durchschnittlichen Ergebnis der physiologischen Teilfragen (Abb. 3b), bei denen die Kontrollgruppe etwas besser abschließen konnte. Die partielle Beweglichkeit der anatomischen Komponenten in konventionellen Modellen im Gegensatz zu den (noch) unbeweglichen Komponenten des VR-Modells scheint das Verständnis der Physiologie zu erleichtern und hier ausschlaggebend zu sein. Dies bestätigt somit die Erkenntnis zur Interaktivität von VR-Modellen, welche von Kyaw et al. bereits als deutlicher Prädiktor für die Vermittlung von Inhalten (z. B. weiterentwickeltes VR-Modell) herausgearbeitet wurde [11]. Auch hier lassen sich bei Ergänzung des VR-Modells durch Visualisierungen von physiologischen und später auch pathologischen Prozessen deutliche Zugewinne erhoffen. Als Beispiele für solche Animation nehme man das Auftreffen von Schallwellen auf das Trommelfell mit der fortleitenden Bewegung der nachgestellten Ossikelkette oder im gleichen Rahmen die Pathologie einer Otosklerose.

Neben physiologischen Aspekten ist aber gerade die komplexe dreidimensionale Anatomie des Mittelohrs bisher mit zweidimensionalen Abbildungen und plastischen Modellen nur schwer vorstell- und erlernbar. Besonders hier wird die Stärke der VR-Technologie gesehen, die nachweislich ein großes Potenzial für Lernprozesse im medizinischen Fachgebiet, z. B. in der chirurgischen Ausbildung, birgt [10, 14]. Izard et al. ergänzen hierbei den Vorteil, Simulationen im Gegensatz zu Übungen mit echten Patienten beliebig oft durchführen können [7]. Eine lernbezogene Anwendung kann – analog zu unserem Studienablauf – in einem begehbaren Raum stattfinden, der Verständnisprozessen dient und als „Explorationswelt“ definiert wird [24]. Dass die Anwendung eines solchen immersiven Modells bei den Studierenden grundsätzlich auf großen Zuspruch stößt, kann mit der sehr guten und abschließend deutlich gesteigerten Haltung zum vorliegenden Konzept gezeigt werden (Abb. 4b). Zudem scheint die prägende Erfahrung in unserem VR-Setting den deutlichen studentischen Wunsch nach weiteren innovativen Lehrmethoden ausgelöst zu haben (*Aussage 8*).

Die Implementierung eines verbesserten VR-Modells soll das traditionelle Lehrkonzept nicht ersetzen, sondern das Lehrangebot und damit die Zugänglichkeit der Inhalte ergänzen und erweitern. Ein solches kombinierendes Konzept von traditioneller und digitalisierter Lehre wurde bereits von vorausgegangen Studien unterstützt. So suggerieren auch die Ergebnisse eines Reviews von Kleinert et al. zu immersiven 3‑D-Patientensimulatoren (IPS) und ihren Einfluss auf den Lernerfolg, dass diese die klinische Lehre nicht ersetzen können, sondern viel mehr Studierende ohne viel klinische Erfahrung unterstützen sollen [8]. Die positive Zukunftsperspektive wird durch die Hypothese einer VR-getriggerten, subjektiv gesteigerten Wissensvermittlung (Abb. 4a) untermauert. Methodisch findet sich dies im Einklang mit Studien zu erworbenen Kompetenzen von Sharaf et al. („ToSkORL“, Teaching of Skills in Otorhinolaryngology), bei welchen eine Übereinstimmung zwischen studentischer Selbst- und standardisiert erfasster Fremdwahrnehmung durch die Dozierenden [25] nachgewiesen wurde.

Ziel einer nachhaltigen Lehre sollte ein effektiver und anhaltender Lerneffekt sein. In dieser Hinsicht stellten sich die Ergebnisse der qualitativen Evaluation besonders eindeutig heraus (*Aussagen 4 und 5*). Aspekte wie eine gesteigerte Nutzungsmotivation bei den Lernenden [16], eine höhere Zufriedenheit, mehr Selbstvertrauen [3] sowie ein grundsätzlich verstärktes erfahrungsbasiertes Lernen [13] wurden bereits in Studien als vorteilhafter Effekt von fortschrittlichen Lehrkonzepten (hier: VR) beschrieben. So zeigte eine Studie von van Bonn et al. zu neuen interaktiven Visualisierungsmöglichkeiten bei Felsenbeinoperationen eine gesteigerte Lerneffizienz und Motivation der Studierenden [28]. Der Lerneffekt wurde in unserer Studie unter der Verwendung der VR-Methode ebenfalls sehr positiv bewertet, auch die Ersterfahrung und das Lernerlebnis mit einer solchen innovativen Lehrmethode, wohingegen kaum negative (technische) Erlebnisse beklagt wurden (*Aussagen 6 und 7*).

Die VR als Lehrmethode birgt einige grundlegende Vorteile: Im Rahmen der Ressourcenoptimierung verschafft ein einzelnes VR-Gerät den Zugang zu verschiedenen Ansichten und Vergrößerungen diverser Modelle und macht diese schlichtweg überflüssig. Im Vergleich dazu wurde der Vergleichsgruppe fünf unterschiedliche Modelle, unter anderem auch ein originales Felsenbeinmodell, zur Verfügung gestellt, um ein Maximum der konventionellen Lehrmöglichkeiten auszuschöpfen. Ein weiterer Vorteil ergibt sich durch die Möglichkeit, die Leistungen und Bewegungen im virtuellen Modell durch evidenzbasierte Metriken zukünftig objektiv zu erfassen und zu bewerten [29].

Allerdings zeigen sich ebenfalls einige Nachteile der VR-Technik. So ist Entwicklung des Modells, die Bereitstellung der Technik und letztendlich die Einweisung und Nutzung von Virtual Reality sehr zeit- und ressourcenintensiv (technische Abhängigkeit). Zudem wird eine mögliche Unterforderung durch Informationsarmut bei immersiven Anwendungen beschrieben [29]. Diese konnten wir beim komplexen Fokus Mittelohr nicht erkennen. Bereits erwähnte Erweiterungen des Modells durch interaktive Komponenten oder ein Einblenden von zusätzlichen Informationen sollten dem auch weiterhin vorbeugen können.

Methodenkritisch ist der zeitliche Faktor der Studie einzuschätzen. Da aufgrund der Corona-Pandemie unter strengen Hygieneauflagen gearbeitet werden musste, war die Zeit der Studierenden an jeder Station des Moduls „Mittelohr“ auf 15 min begrenzt. In dieser Zeit musste nach Möglichkeit ebenfalls die Beantwortung des Fragebogens abgeschlossen werden. Weitere Kritikpunkte könnten ein fehlender Pre-Test zum besseren Gruppenvergleich und eine stringentere Ausrichtung des Fragebogens der formativen Prüfung auf anatomisch-räumliche Aspekte sein.

Ein wichtiger Aspekt beim Einsatz digitalisierter Lehre im Rahmen der digitalen Transformation ist ein steter Anpassungsprozess. Dabei sollten nach dem Prinzip „agility by design“ bereits bei der Konzeption derartiger Curricula gewisse Freiräume geschaffen werden, die eine iterative Anpassung im laufenden Lehrbetrieb ermöglichen [2]. Hierfür ist eine qualitative und quantitative Evaluation neu implementierter Formate essenziell. Die Implementierung und Weiterentwicklung der VR bleibt somit eine fortwährende Herausforderung.

## Supplementary Information





## References

[1] Albrecht T, Nikendei C, Praetorius M (2021). Face, content, and construct validity of a virtual reality otoscopy simulator and applicability to medical training. Otolaryngol Head Neck Surg.

[2] Arbeitsgruppe Curriculum 4.0. (2018) Curriculumentwicklung und Kompetenzen für das digitale Zeitalter – Thesen und Empfehlungen der AG Curriculum 4.0 des Hochschulforum Digitalisierung. Arbeitspapier Nr. 39. Hochschulforum Digitalisierung. Berlin

[3] Bakhos D, Galvin J, Aoustin J-M, Robier M, Kerneis S, Bechet G, Montembault N, Laurent S, Godey B, Aussedat C (2020). Training outcomes for audiology students using virtual reality or traditional training methods. PLoS ONE.

[4] Dombrowski T, Dazert S, Volkenstein S (2019). Digitale Strategien in der Lehre. Laryngorhinootologie.

[5] Freiherr von Saß P, Klenzner T, Scheckenbach K, Chaker A (2017). Einsatz von E-Learning an deutschen Universitäts-HNO-Kliniken. Laryngorhinootologie.

[6] http://hno-lernprogramm.uniklinik-freiburg.de. Zugegriffen: 8. Jan. 2022

[7] Izard SG, Juanes JA, García Peñalvo FJ, Estella JMG, Ledesma MJS, Ruisoto P (2018). Virtual reality as an educational and training tool for medicine. J Med Syst.

[8] Kleinert R, Wahba R, Chang D-H, Plum P, Hölscher AH, Stippel DL (2015). 3D immersive patient simulators and their impact on learning success: a thematic review. J Med Internet Res.

[9] Krauss F, Giesler M, Offergeld C (2021). Zur Effektivität der digitalen Vermittlung praktischer Fertigkeiten in der curricularen HNO. Lehre. HNO.

[10] Kuhn S, Huettl F, Deutsch K, Kirchgässner E, Huber T, Kneist W (2021). Chirurgische Ausbildung im digitalen Zeitalter – Virtual Reality, Augmented Reality und Robotik im Medizinstudium. Zentralbl Chir.

[11] Kyaw BM, Saxena N, Posadzki P, Vseteckova J, Nikolaou CK, George PP, Divakar U, Masiello I, Kononowicz AA, Zary N, Car TL (2019). Virtual reality for health professions education: systematic review and meta-analysis by the digital health education collaboration. J Med Internet Res.

[12] Lang F, Everad B, Knopf A, Kuhn S, Offergeld C (2021). Digitalisierung in der curricularen Lehre: Erfahrungen mit dem Freiburger HNO-Lernprogramm. Laryngorhinootologie.

[13] Lerner D, Wichmann D, Wegner K (2019). Virtual-Reality-Simulationstraining in der Notfallsanitäterausbildung. retten!.

[14] Maresky HS, Oikonomou A, Ali I, Ditkofsky N, Pakkal M, Ballyk B (2019). Virtual reality and cardiac anatomy: exploring immersive three-dimensional cardiac imaging, a pilot study in undergraduate medical anatomy education. Clin Anat.

[15] Medizinischer Fakultätentag der Bundesrepublik e. V. Nationaler Kompetenzbasierter Lernzielkatalog – NKLM 2.0. 2021.

[16] Moro C, Birt J, Stromberga Z, Phelps C, Clark J, Glasziou P, Scott AM (2021). Virtual and augmented reality enhancements to medical and science student physiology and anatomy test performance: a systematic review and meta-analysis. Anat Sci Educ.

[17] Offergeld C, Ketterer M, Neudert M, Hassepaß F, Weerda N, Richter B, Traser L, Becker C, Deeg N, Knopf A, Wesarg T, Rauch A-K, Jakob T, Ferver F, Lang F, Vielsmeier V, Hackenberg S, Diensthuber M, Praetorius M, Hofauer B, Mansour N, Kuhn S, Hildenbrand T (2021). „Ab morgen bitte online“: Vergleich digitaler Rahmenbedingungen der curricularen Lehre an nationalen Universitäts-HNO-Kliniken in Zeiten von COVID-19 : Digitale Lehre an nationalen Universitäts-HNO-Kliniken. HNO.

[18] Offergeld C, Neudert M, Emerich M, Schmidt T, Kuhn S, Giesler M (2020). Vermittlung digitaler Kompetenzen in der curricularen HNO-Lehre: abwartende Haltung oder vorauseilender Gehorsam?. HNO.

[19] Offergeld C, Praetorius M, Neudert M (2020). „ENT—expect no teaching“ …?! : Welchen Stellenwert haben Aus‑, Weiter- und Fortbildung in der deutschen HNO-Heilkunde?. HNO.

[20] Offergeld C, von Schnakenburg P, Knopf A, Pfirrmann P, Kromeier J, Heermann S (2021). Innovation trifft Tradition: Virtual Reality. VR) in der HNO-Lehre. Laryngorhinootologie.

[21] Pather N, Blyth P, Chapman JA, Dayal MR, Flack NAMS, Fogg QA, Green RA, Hulme AK, Johnson IP, Meyer AJ, Morley JW, Shortland PJ, Štrkalj G, Štrkalj M, Valter K, Webb AL, Woodley SJ, Lazarus MD (2020). Forced disruption of anatomy education in Australia and New Zealand: an acute response to the Covid-19 pandemic. Anat Sci Educ.

[22] Polk M-L, Lailach S, Kemper M, Bendas A, Zahnert T, Neudert M (2020). Lernkurve der HNO-Spiegeluntersuchung : Zielgerichtete Lehrveranstaltungsplanung zu einer psychomotorischen Fertigkeit. HNO.

[23] de Ponti R, Marazzato J, Maresca AM, Rovera F, Carcano G, Ferrario MM (2020). Pre-graduation medical training including virtual reality during COVID-19 pandemic: a report on students’ perception. BMC Med Educ.

[24] Schwan S, Buder J (2006). Virtuelle Realität und E-Learning.

[25] Sharaf K, Felicio-Briegel A, Widmann M, Huber J, Eggersmann TK, Stadlberger U, Schrötzlmair F, Canis M, Lechner A (2021). ToSkORL: Selbst- und Fremdeinschätzung bei der Untersuchung des Kopf-Hals-Bereichs. HNO.

[26] Stepan K, Zeiger J, Hanchuk S, Del Signore A, Shrivastava R, Govindaraj S, Iloreta A (2017). Immersive virtual reality as a teaching tool for neuroanatomy. Int Forum Allergy Rhinol.

[27] Tolks D, Schäfer C, Raupach T, Kruse L, Sarikas A, Gerhardt-Szép S, Kllauer G, Lemos M, Fischer MR, Eichner B, Sostmann K, Hege I (2016). An introduction to the inverted/flipped classroom model in education and advanced training in medicine and in the healthcare professions. GMS J Med Educ.

[28] van Bonn SM, Grajek JS, Schuldt T, Schraven SP, Schneider A, Rettschlag S, Oberhoffner T, Weiss NM, Mlynski R (2022). Interaktive intraoperative Annotation chirurgischer Anatomie in der studentischen Ausbildung zur Unterstützung der Lerneffizienz und -motivation. HNO.

[29] Wannemacher K, Jungermann I, Scholz J, Tercanli H, Villiez A (2016). Digitale Lernszenarien im Hochschulbereich.

