# Supplementary figures and images for: Use of virtual reality in ENT teaching: an alternative to the conventional anatomic model
Source: HNO. 2022 Dec 7;71(2):106–13. [Article in German] doi: 10.1007/s00106-022-01252-z (PMC9734900; doi:10.1007/s00106-022-01252-z)

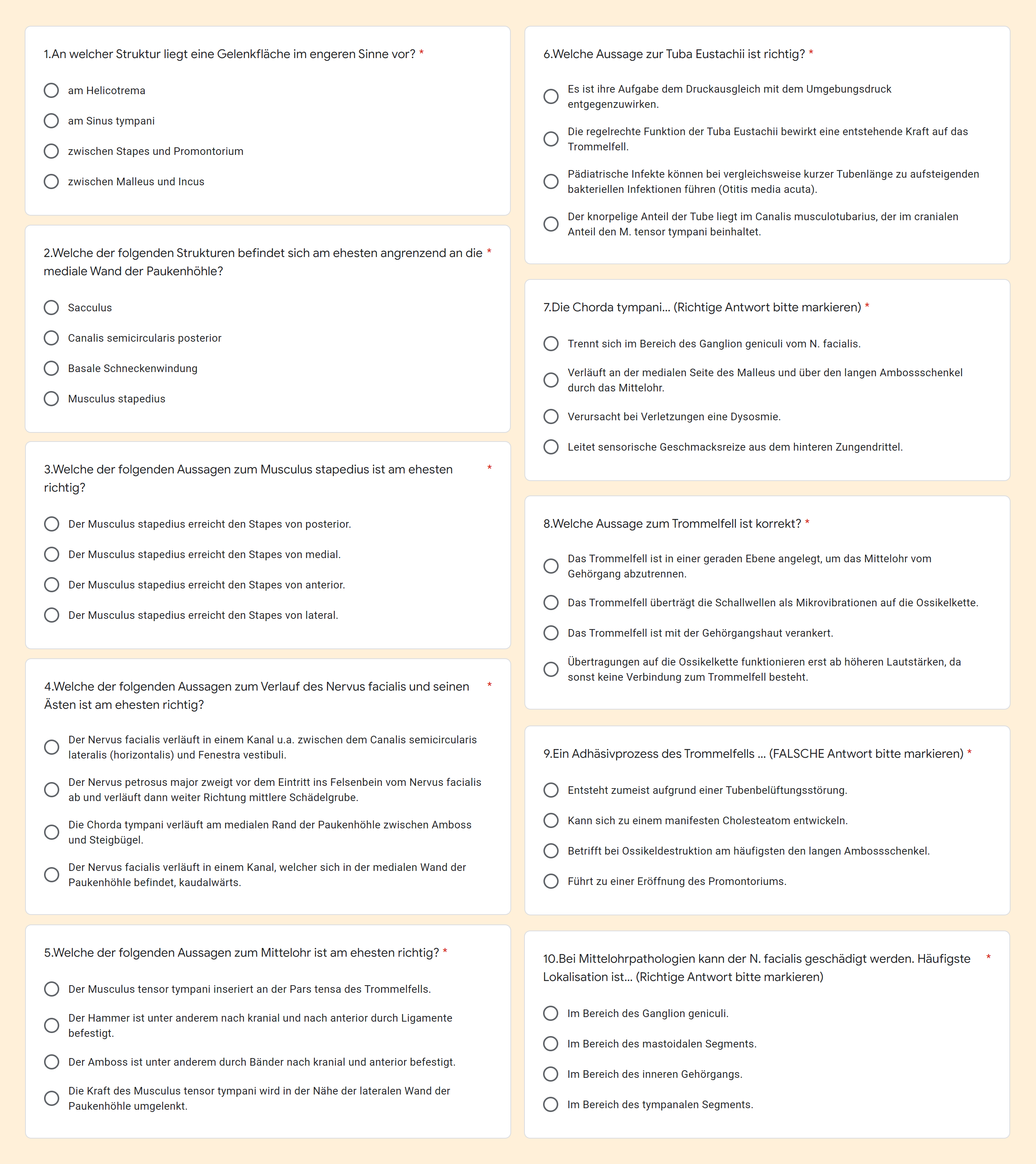

Supplement: Supplementary file 1 [file 106_2022_1252_MOESM1_ESM.tif]
